# Hybrid model for ecological vulnerability assessment in Benin

**DOI:** 10.1038/s41598-021-81742-2

**Published:** 2021-01-28

**Authors:** Jacqueline Fifame Dossou, Xu Xiang Li, Mohammed Sadek, Mohamed Adou Sidi Almouctar, Eman Mostafa

**Affiliations:** Department of Earth & Environmental Science, Institute of Global Environmental Change, School of Human Settlements and Civil Engineering, Xi’an Jiaotong University, Xi’an, 710049 China

**Keywords:** Climate sciences, Ecology, Environmental sciences, Natural hazards

## Abstract

Identifying ecologically fragile areas by assessing ecosystem vulnerability is an essential task in environmental conservation and management. Benin is considered a vulnerable area, and its coastal zone, which is subject to erosion and flooding effects, is particularly vulnerable. This study assessed terrestrial ecosystems in Benin by establishing a hybrid ecological vulnerability index (EVI) for 2016 that combined a composite model based on principal component analysis (PCA) with an additive model based on exposure, sensitivity and adaptation. Using inverse distance weighted (IDW) interpolation, point data were spatially distributed by their geographic significance. The results revealed that the composite system identified more stable and vulnerable areas than the additive system; the two systems identified 48,600 km^2^ and 36,450 km^2^ of stable areas, respectively, for a difference of 12,150 km^2^, and 3,729 km^2^ and 3,007 km^2^ of vulnerable areas, for a difference of 722 km^2^. Using Moran’s I and automatic linear modeling, we improved the accuracy of the established systems. In the composite system, increases of 11,669 km^2^ in the potentially vulnerable area and 1,083 km^2^ in the highly vulnerable area were noted in addition to a decrease of 4331 km^2^ in the potential area; while in the additive system, an increase of 3,970 km^2^ in the highly vulnerable area was observed. Finally, southern Benin was identified as vulnerable in the composite system, and both northern and southern Benin were identified as vulnerable in the additive system. However, regardless of the system, Littoral Province in southern Benin, was consistently identified as vulnerable, while Donga Province was stable.

## Introduction

The world is currently facing a disruption in the climate system that is resulting in global warming. According to the fourth assessment report (AR4) of the Intergovernmental Panel on Climate Change (IPCC), the average rate of warming over the last fifty (50) years, on the order of 0.13 °C per decade, has nearly doubled compared to that in the last 100 years. Climate alteration^1,2^ implies severe drastic repercussions for ecosystems and disturbances to diversity. Therefore, climate change, in addition to being an environmental issue, is generating a global consensus regarding the effects of global warming in different activity sectors and is now a development concern, especially with regard to sustainable development^3^. Indeed, the impacts of global warming do not spare any sector of human development or any ecosystem, whether marine, desert, forest, aquatic or terrestrial. Land degradation, which decreases the productive capacity of soils, is one of the major problems for the future of an increasingly anthropogenic planet, particularly in developing countries that are experiencing ever-increasing demographic pressure. It is therefore reasonable that this problem is of great concern to scientific authorities. Ecological vulnerability, one way to evaluate the status of an ecosystem, is highly dependent on climate change because climate data are a key factor in ecological vulnerability assessments. Recently, the ecological vulnerability concept has been studied and applied in several disciplines^4,5^. The main objective of ecological vulnerability research is to maintain the balance between protection and exploitation that is vital for the sustainability of an ecosystem by gradually identifying vulnerable areas; these areas may then become the subject of special attention^6^. Delimiting ecologically vulnerable zones is a fundamental aspect of environmental conservation management^7^.

Africa, because of its development level, is often described as one of the most vulnerable regions in the world^8^. Overall, research studies have concluded that the main consequences of climate change in West Africa are coastal erosion, floods, drought, lack of access to drinking water for approximately two hundred (200) million people^9^, etc. Benin, which is a part of West Africa, has recorded a drastic 20 to 40% decrease in major river flows since the 1970s^10^. Rainfall in Benin is projected to continue to decrease by 10 to 20% compared to current levels by 2025^11^. In Benin, the land degradation situation is of great concern at the national level. According to the default data provided by the National Remote Sensing Center, it is estimated that in 2016, approximately 2.2 million hectares of land, or 19% of the national territory, were degraded^10,12^. In addition, longer periods of drought and more intense rainy seasons are expected in Benin^13,14^. Southern Benin, specifically Littoral Province, is the location of most administrative offices as well as the largest market in Benin (Dantokpa), which is also one of the largest markets in the subregion. As a result, it has become the residence of many public officials and businesspeople. To meet these housing needs, some natural water drainage channels have been filled, which has hindered the flow of water and made the area vulnerable to flooding.

To face all these challenges, Benin must develop mechanisms for prevention, mitigation and adaptation. Since the stakes are high, decisions made today will determine the living conditions of future generations. In this context, the main objective of our study is to assess the ecological vulnerability of Benin’s terrestrial ecosystems to climate change. Ecological assessments of ecosystems are becoming essential^15^ for both understanding an ecological zone and for developing it. Vulnerability is an indicator that incorporates several variables and attributes^16^. It is useful to have a good understanding of the different patterns of spatial variation in an area. Spatial assessment is beneficial because it can be used to display complex data in simple and visually appealing ways^17^; this can also be a weakness because uncertainties in the data and important analytic assumptions that affect the output maps are often hidden from the user. The ecological vulnerability index (EVI), which is an important tool for environmental assessment, was recently developed^18,19^. This index concept has been studied and applied at different spatial scales and in several regions^16,20–23^. To date, studies have developed established systems based on various techniques^24^ such as the fuzzy evaluation method, the gray evaluation method, principal component analysis (PCA), the artificial neural network evaluation method, the landscape evaluation method, and the analytic hierarchy process (AHP) method^25^, to evaluate the EVI^26^. Ecological vulnerability^27,28^ is a universal term that can be used at many levels (site, ecosystem, community, overall environment, etc.) and, referring to the IPCC^9^, is also considered an additive function of exposure to a stressor, sensitivity to the stressor and adaptation, which can be interpreted as resilience^29,30^.

Research on ecological vulnerability is crucial or climate change mitigation and plays a key role in revealing where how and why ecosystems are affected. The importance of such studies is now widely recognized, as they have been justified by observed and projected climate conditions that highlight the urgency of understanding the implications of a rapidly changing climate^24,31^. The purpose of this study is to help understand terrestrial ecosystems in the Republic of Benin by assessing their vulnerability to climate change in order to accurately identify fragile ecological areas within those ecosystems. We adopted different conventional frameworks to better understand the ecological state of Benin’s terrestrial ecosystems. Relying on existing data, we assessed Benin’s ecological vulnerability to climate by establishing a hybrid ecological vulnerability index for 2016. Using fifteen indicators, we calculated the ecological vulnerability index for terrestrial ecosystems in Benin based first on principal component analysis (PCA) and second on the additive method recommended by the IPCC. We then determined the spatial distribution^25,32^ of the EVI values to provide an exhaustive analysis. The spatial variation results were mapped to reveal the points of discrepancy between the two established systems. The comparative analysis focused on fluctuations in the vulnerability ranking to facilitate the understanding of the interactions among the constituent components and to provide assessment results with respect to the various attributes considered in the index. Finally, based on automatic linear modeling, we improved the accuracy of our established systems by improving our classification system. The study components were normalized to a commensurate scale from 0 to 1 from lowest least to highest to eliminate any other effects of the normalization and weightings on the outcomes. As recommended by the IPCC in its AR5 report, these indicators were selected by consulting experts and existing studies that noted some factors, such as climate, environmental hazard, and socioeconomic factors, as threats to ecosystems in Benin.

## Description of the study area

**Figure 1:**
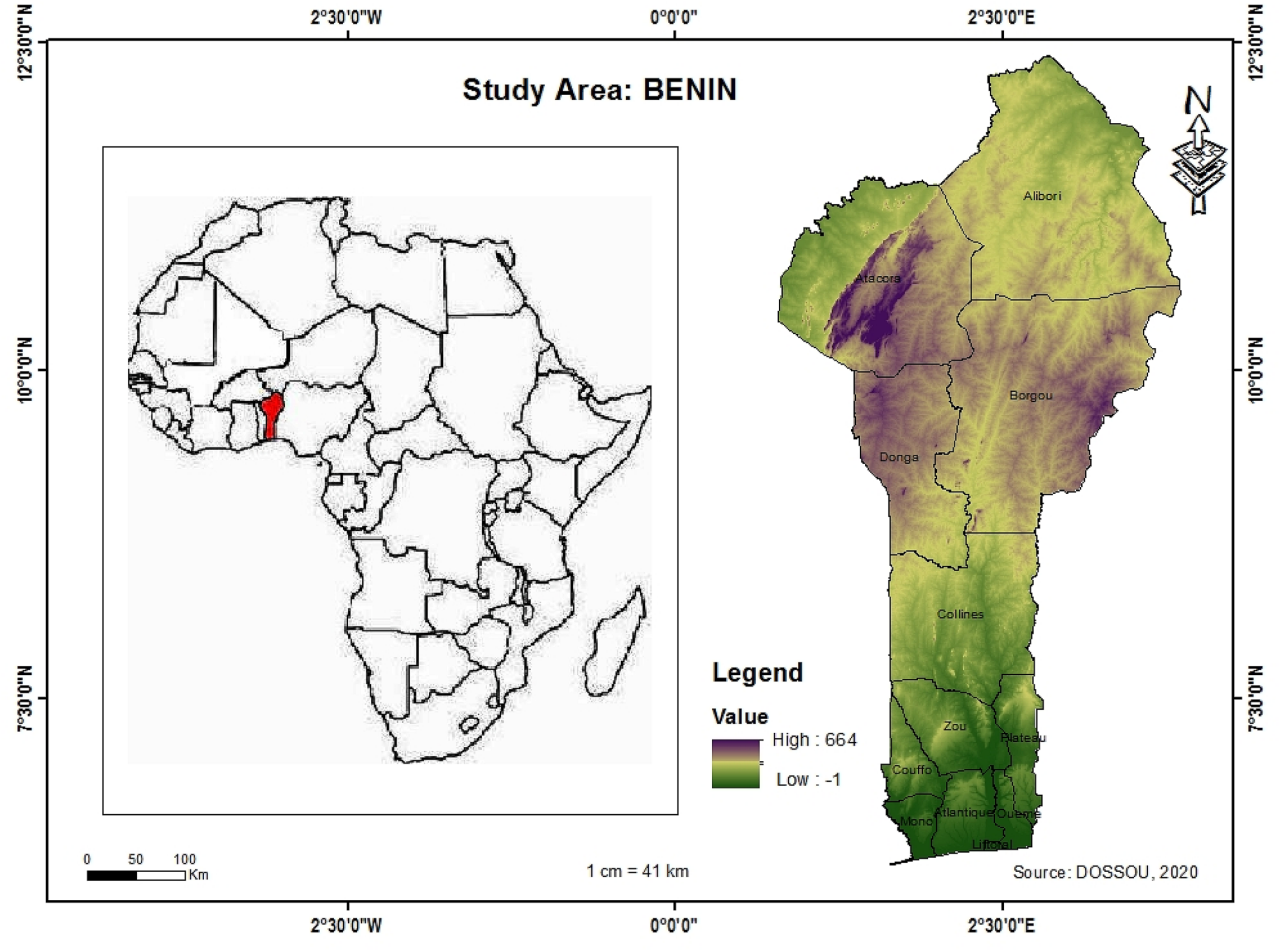
Location of study area.

Benin is located in West Africa between 6°10′ and 12°25′ north latitude and between 0°45′ and 3°55′ east longitude. It covers an area of 114,763 square kilometers (km^2^), and currently has 12 provinces (Fig. 1). The area of potential cultivable land is estimated to be approximately 7 million hectares, i.e., nearly 63% of the total area, and some forests are classified as being exposed to degradation. Benin is characterized by two well-defined climatic zones: the southern zone, which has a subequatorial climate with two rainy seasons per year, and the northern zone, which has a continental tropical climate with one rainy season. These zones are separated by a transition zone. Central Benin has a transitional climate similar to the sub-Sudanese climate. Benin receives between 700 and 1300 mm of rainfall per year that is spread over 70 to 110 days of the year. This rainfall is characterized by wide spatial and temporal variations, making the cultivation of rain-fed crops particularly unpredictable. The average maximum temperatures throughout the country fluctuate between 28 and 33.5 °C, while the average minimum temperatures range from 24.5 to 27.7 °C. Currently, the total population is estimated at 10,700,000 inhabitants, and the per capita density varies between 31 inhabitants per km^2^ in the province of Alibori (northern Benin) and 10,160 inhabitants per km^2^ in the province of Littoral (southern Benin)^10,12,33^, the highest-density province. Littoral is the only province with a single municipality and is also the economic capital of the country.

## Methodology

### Data and processing

#### Establishment of the ecological vulnerability index (EVI) system

Vulnerability is an indicator that incorporates several multidimensional and multivariate attributes^7,34,35^. Given the objective of this study, it was considered better to rely on freely available data or data that could be generated within the study, such as slope and elevation, which were generated through the use of a digital elevation model (DEM) by applying a geographic information system (GIS) associated with remote sensing (RS) (Table 1). The current study considered the EVI to be a function of fifteen indicators. These include climate data^4,18,25^ such as rainfall, temperature, relative humidity, and sunlight; socioeconomic data^36–38^ such as population, density per inhabitant, gross domestic product (GDP), and number of houses; thematic data such as the normalized difference vegetation index (NDVI), soil organic carbon, digital elevation model (DEM) and slope; and environmental hazard data^3,34^ such as flood, drought and bush fire frequency. Climate data were obtained from the Agency for Aerial Navigation Safety in Africa and Madagascar (AANSAM), socioeconomic data were provided by the National Institute for Statistics and Economic Analysis (NISEA), and hazard data were obtained from the United Nations Environment Program (UNEP) platform (https://preview.grid.unep.ch). Raster data were sampled using the bilinear method, which determines the new value of a cell based on the weighted average of the distance between the four nearest input cell centers. Then, all data were processed using ArcGIS 10.5 and SPSS 21.

**Table 1:** Characteristic of data. mm = millimeter, ºC = degree Celsius, % = percentage, h/m^2^ = hour per meter square, Inh. = inhabitant, Inh/km^2^ = inhabitant per kilometer square, FCFA = francs des colonies françaises d’Afrique, AANSAM = Agency for Aerial Navigation Safety in Africa and Madagascar, and NISEA = National Institute for Statistics and Economic Analysis.

Group	Indicators	Units	Period	Resolution	Processing	Source (Ref)
Climate	Rainfall Temperature R.Hunidity Sunlight	Mm ºC % h/m^2^	2000–2016	Points data	IDW Interpolation method and resampling	AANSAM
Socio-economic	Population Density GDP Houses	Inh Inh/km^2^ FCFA Houses				NISEA
Thematic	NDVI	–	2016	250 m	Resampling	https://earthexplorer.usgs.gov/
	Elevation Slope	–	2016	90 m	Resampling	Digital elevation model
	Soil organic carbon	–	2014	1 km	Resampling	https://soilgrids.org/#!/?layer=ORCDRC_M_sl2_250m&vector=1
Hazard	Flood Drought Fire	–	2013	1 km	Resampling	https://preview.grid.unep.ch

### Method

Mapping degraded lands emphasizes the gradual changes in the spatial distribution of degradation^39^, which is determined based on ecological vulnerability assessments. To assess ecological vulnerability in this study^3^, we complied a dataset of the fifteen indicators mentioned above. The EVI was established in two different ways. First, PCA was applied to all fifteen indicators to determine their relative degree of influence, which was reflected in their weighting coefficient. Second, based on the IPCC’s recommendation, vulnerability was determined as an additive function of exposure, sensitivity and adaptation. PCA was used to obtain the weighted coefficients by extraction, and then each indicator was multiplied by its extraction coefficient to obtain the variance rate, as shown in Table 2. The point data were transformed into raster data using inverse distance weighted (IDW) interpolation. In a vulnerability assessment, it is important to accurately determine the assessment indicator weights. We checked the spatial autocorrelation of the outcomes using Moran’s I and cluster analysis.

**Table 2:** PCA weight based on eigenvalues and variance.

Indicators	Initial Eigenvalues			Extraction sums of squared loadings		
	Total	% of Variance	Cumulative percentage %	Total	% of Variance	Cumulative percentage %
1	5.479	36.525	36.525	5.479	36.525	36.525
2	1.951	13.009	49.534	1.951	13.009	49.534
3	1.518	10.117	59.651	1.518	10.117	59.651
4	1.269	8.462	68.113	1.269	8.462	68.113
5	1.138	7.587	75.700	1.138	7.587	75.700
6	0.937	6.248	81.948	0.937	6.248	81.948

#### Inverse distance weighted (IDW) interpolation

IDW interpolation is an accurate method that ensures that the estimated value of a point is more influenced by closer identified points than by more distant ones^40^. IDW assumes that the correlation degree and similarity between variables are relative to the distance between them, which can be interpreted as an inverse distance. The general equation for the IDW method is shown in Eq. (1):

1 $$ Zo = \frac{{\mathop \sum \nolimits_{i = 1}^{N} zi.d_{i}^{ - n} }}{{\mathop \sum \nolimits_{i = 1}^{N} d_{i}^{ - n} }} $$

z_0_ = estimated value of variable z at point i, z_i_ = sample value at point i, d_i_ = distance of a sample point to an estimated point, N = coefficient that determines the weight based on distance, n = total number of predictions for each validation case.

#### Composite system: principal component analysis

A very common application of PCA is to produce a summary of uncorrelated variables from multivariable information^7,34,35^, since the objective of PCA is to reduce data dimensionality by extracting the maximum information along linear axes called principal components.

The multiple vulnerability indicators combined in the aggregate vulnerability index exceeded the individual measurement units of each indicator. For this reason, PCA requires that the study variables be ranked on the same unit scale^38^. Therefore, it was essential that these variables be standardized. We chose to use a scaled standardization ranking from 0 to 1 by applying the formula below to each indicator Eq. (2):

2 $$ {\text{Vij}} = \frac{{{\text{v}}_{{{\text{ij}}}} - {\text{v}}_{{{\text{minj}}}} }}{{{\text{v}}_{{{\text{maxj}}}} - {\text{v}}_{{{\text{minj}}}} }} $$

where ${\text{Vij}}$ represents the standardized value of factor j of grid i, ranked from 0 to 1, ${\text{v}}_{{{\text{ij}}}}$ represents the measured value of factor j of grid i, and ${\text{v}}_{{{\text{minj}}}}$ and ${\text{v}}_{{{\text{maxj}}}}$ represent the minimum and maximum values of factor j of grid i, respectively.

Once all data were resampled and standardized, the study area was subdivided into a fishnet polygon of 1 km per grid to extract the real value of each studied indicator variable at each pixel level size. Then, the EVI was established using PCA in SPSS 21 software to calculate the weighted coefficient of each indicator^41^.

##### Spatial principal component analysis

In a PCA, the main objective is to reduce a set of *p* variables to a set of uncorrelated linear variables called principal components^18,37^. Transforming all data into an integrated assessment index is fundamental to performing an ecological vulnerability assessment but remains a difficult task to achieve. Referring to Eq. (3), the principal components can be expressed as follows:

3 $$ {\text{PCn}} = {\text{w}}_{{{\text{i1}}}} {\text{x}}_{{1}} + {\text{wi}}_{{2}} {\text{x}}_{{2}} + \cdots + {\text{w}}_{{{\text{ip}}}} {\text{x}}_{{\text{p}}} $$

where PCn is the principal component score, w is the component loading, x is the measured value of a variable, i is the component number and *p* is the total number of variables.

As shown in Eq. (4), each principal component is multiplied by its variance rate.

4 $$ {\text{EVI}}_{{{\text{PCA}}}} = {\text{ r}}_{{1}} {\text{PC}}_{{1}} + {\text{ r}}_{{2}} {\text{PC}}_{{2}} + \cdots + {\text{ r}}_{{\text{q}}} {\text{PC}}_{{\text{q}}} = \mathop \sum \limits_{1}^{6} {\text{PC}} $$

where EVI is the ecological vulnerability index, r is the contribution ratio of the principal component, PC is the principal component, q is the number of principal components retained, n = 6 and, referring to Eq. (5), the coefficient **r** is defined as:

5 $$ {\text{ri}} = { }\frac{{{\text{bi}}}}{{\mathop \sum \nolimits_{{{\text{i}} = 1}}^{{\text{p}}} {\text{bi}}}} $$

where r_i_ is the contribution ratio of the ith principal component and b_i_ is the eigenvalue of the ith principal component.

#### Additive system

The IPCC AR5 report^9^, entitled the Vulnerability Reference Guide, on pages 21 and 22 of the French translation, defines exposure as the nature and degree to which a system under degradation is facing significant climate change; sensitivity is defined as the degree to which a system is affected negatively or positively; and adaptation is defined as the state of system recovery as reflected in its ability to self-regulate against climate change effects^14^. On page 21, this guide also notes that among all elements that contribute to vulnerability, exposure is the most directly related to climate factors^9,41^. Fire^34,42^ is a natural process that has played a fundamental role in maintaining natural ecosystems for millions of years and that regulates the dynamics of plant and animal populations. Moreover, people use fire as a means of cleaning farms for the next planting season. Thus, we classified it as an adaptation indicator. GDP is also presented as an adaptation indicator on page 67 of that guide. Based on these assumptions, our fifteen indicators were grouped into the following three categories: exposure (Eq. 6a; temperature, rainfall, relative humidity, sunlight, flood and drought), sensitivity (Eq. 6b; population, density per inhabitant, elevation, slope, NDVI, soil organic carbon and number of houses), and adaptation (Eq. 6c; GDP and fire).

6 $$ \begin{aligned} {\text{Vulnerability}} & = {\text{f }}\left( {{\text{exposure}},\;{\text{sensitivity}},\;{\text{adaptation}}} \right) \\ {\text{Vulnerability}} & = { }\frac{{{\text{exposure}},\,{\text{sensitivity}},\;{\text{adaptation}}}}{3} \\ \end{aligned} $$

With:

6a $$ \begin{aligned} {\text{Exposure}} & = {\text{f }}\left( {{\text{temperature}},\;{\text{rainfall}},\,{\text{relative}}\;{\text{humidity}},\;{\text{sunlight}},\;{\text{flood}},\;{\text{drought}}} \right) \\ {\text{Exposure}} & = { }\frac{{{\text{temperature}} + {\text{rainfall}} + {\text{relative humidity}} + {\text{sunlight}} + {\text{flood}} + {\text{drought}}}}{6} \\ \end{aligned} $$

6b $$ \begin{aligned} {\text{Sensitivity}} & = {\text{f }}\left( {{\text{population}},\;{\text{density}},\;{\text{elevation}},\,{\text{slope}},\;{\text{NDVI}},\;{\text{organic}}\;{\text{carbon}},\;{\text{houses}}} \right) \\ {\text{Sensitivity}} & = { }\frac{{{\text{population}} + {\text{density}} + {\text{elevation}} + {\text{slope}} + {\text{NDVI}} + {\text{organic }}\;{\text{carbon}} + {\text{houses}}}}{7} \\ \end{aligned} $$

6c $$ \begin{aligned} {\text{Adaptation}} & = {\text{f }}\left( {{\text{GDP}},\;{\text{fire}}} \right) \\ {\text{Adaptation}} & = { }\frac{{{\text{GDP}} + {\text{fire}}}}{2} \\ \end{aligned} $$

## Results

### Composite ecological vulnerability index (EVI_PCA_)

**Figure 2:**
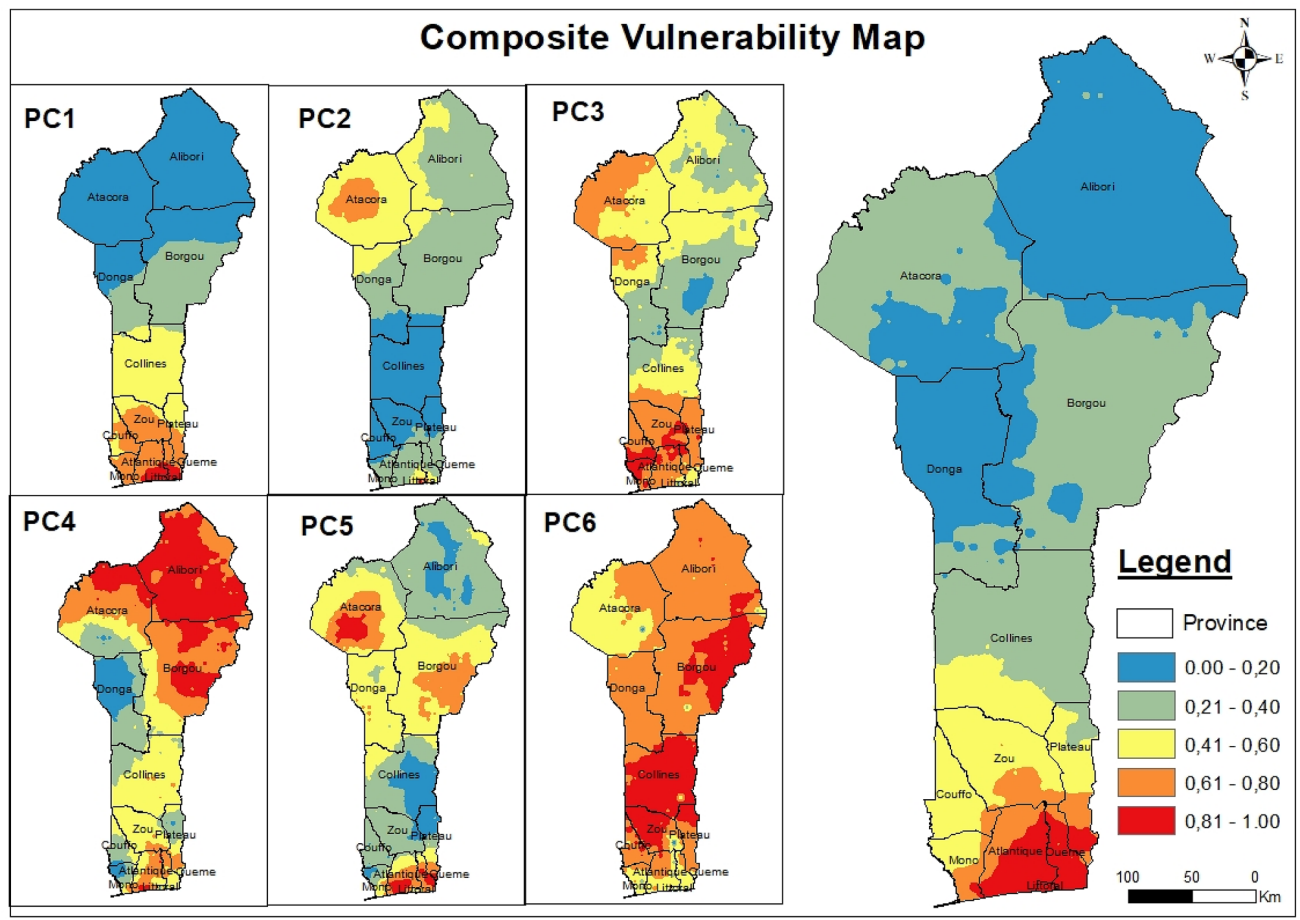
Composite vulnerability map.

We assumed that the system consistency was strong when the cumulative percentage of the extracted principal components was higher than 80% (the cumulative percentage is the quantitative extraction rate of the indicators under study). Six of the principal components met this requirement and cumulatively accounted for 81.948% of the variance. The extraction rates of the individual principal components expressed as the percentage of the variance they explained were PC1 = 36.525%, PC2 = 13.009%, PC3 = 10.117%, PC4 = 8.462%, PC5 = 7.587% and PC6 = 6.248, Table 2; more details about the retained principal components are provided in Table 3. Next, the EVI_PCA_ was calculated using Eq. (4). As shown in Table 4, using equal intervals, the EVI was divided into five (05) classes, namely, potential, slight, low, moderate and high. According to Fig. 2, the composite EVI varied spatially from north to south, and the vulnerability increased from north to south. In southern Benin, the vulnerability conditions were critical; this was especially true in Littoral Province, the economic capital of Benin, which had the highest per capita density at more than 10,000 inhabitants per square kilometer in 2016 according to the collected data, and in Atlantique and Oueme Provinces, which are newly developed residential areas. Alibori Province was determined to be the most stable (potentially vulnerable, according to the classification in Table 4), the least affected, the most spacious and the least occupied area, with a density per capita of approximately 30 inhabitants per square kilometer.

**Table 3:** Retained principal components constituents rate.

Indicators	PC1	PC2	PC3	PC4	PC5	PC6
Humidity	0.927	− 0.072	0.248	− 0.196	0.093	− 0.011
Sunlight	− 0.868	0.203	− 0.222	0.255	− 0.089	− 0.029
Houses	0.849	− 0.093	− 0.059	0.416	0.252	0.032
Fire	− 0.808	0.363	0.054	0.276	0.016	− 0.078
Density	0.751	0.515	− 0.090	− 0.017	0.020	0.058
Elevation	− 0.743	0.025	− 0.242	− 0.181	0.347	0.170
Temperature	0.680	− 0.481	− 0.049	0.132	-0.417	0.170
NDVI	− 0.543	0.247	0.459	0.262	− 0.056	0.085
Drought	0.347	0.631	− 0.264	− 0.130	− 0.163	0.321
GDP	0.237	0.592	− 0.297	− 0.099	− 0.253	0.333
Rainfall	0.373	0.567	0.484	− 0.203	0.384	− 0.239
Carbon	− 0.022	0.144	0.617	0.398	− 0.452	0.140
Population	0.380	0.029	− 0.361	0.726	0.399	0.090
Slope	− 0.125	− 0.176	0.459	− 0.023	0.398	0.644
Flood	0.376	0.288	0.084	0.207	− 0.018	− 0.383

**Table 4:** References for classification. *Note* The scale^32,41^ 0–1 indicating the lowest EVI level (0) to the highest EVI level (1)is used.

Vulnerability rank	Vulnerability classification	Classification description
0.00–0.20	Potential	Stable ecosystem, rich soil and good vegetation cover
0.21–0.40	Slight	Relatively stable ecosystem, rich soil, relatively good vegetation cover
0.41–0.60	Low	Relatively stable ecosystem, infertile soil, relatively poor vegetation cover
0.61–0.80	Moderate	unstable ecosystem, bad quality soil, poor vegetation cover
0.81–1.00	High	Extremely unstable ecosystem, deteriorated soil, extremely poor vegetation

To summarize, the EVI_PCA_ results revealed potential vulnerability in Alibori and Donga provinces, slight vulnerability in Atacora, Borgou and Collines provinces, low vulnerability in the northern Zou, Plateau, Mono and Couffo provinces, moderate vulnerability in southern Zou, Plateau Mono, Couffo and northern Atlantique and high vulnerability in Littoral, Oueme and southern Atlantique provinces. Littoral and parts of Atlantique and Oueme provinces were identified as extremely vulnerable areas. Each classified area was mapped in Fig. 4. It is important to highlight that even at 75%, 80% and 85% cumulative percentages, the spatial variations remained the same.

$$ {\text{EVI}}_{{{\text{PCA}}}} = 0.{446} \times {\text{PC1}} + 0.{159} \times {\text{PC2}} + 0.{123} \times {\text{PC3}}_{ + } 0.{1}0{3} \times {\text{PC4}} + 0.0{93} \times {\text{PC5}} + 0.0{75} \times {\text{PC6}} $$

### Additive ecological vulnerability index (EVI_ad_)

**Figure 3:**
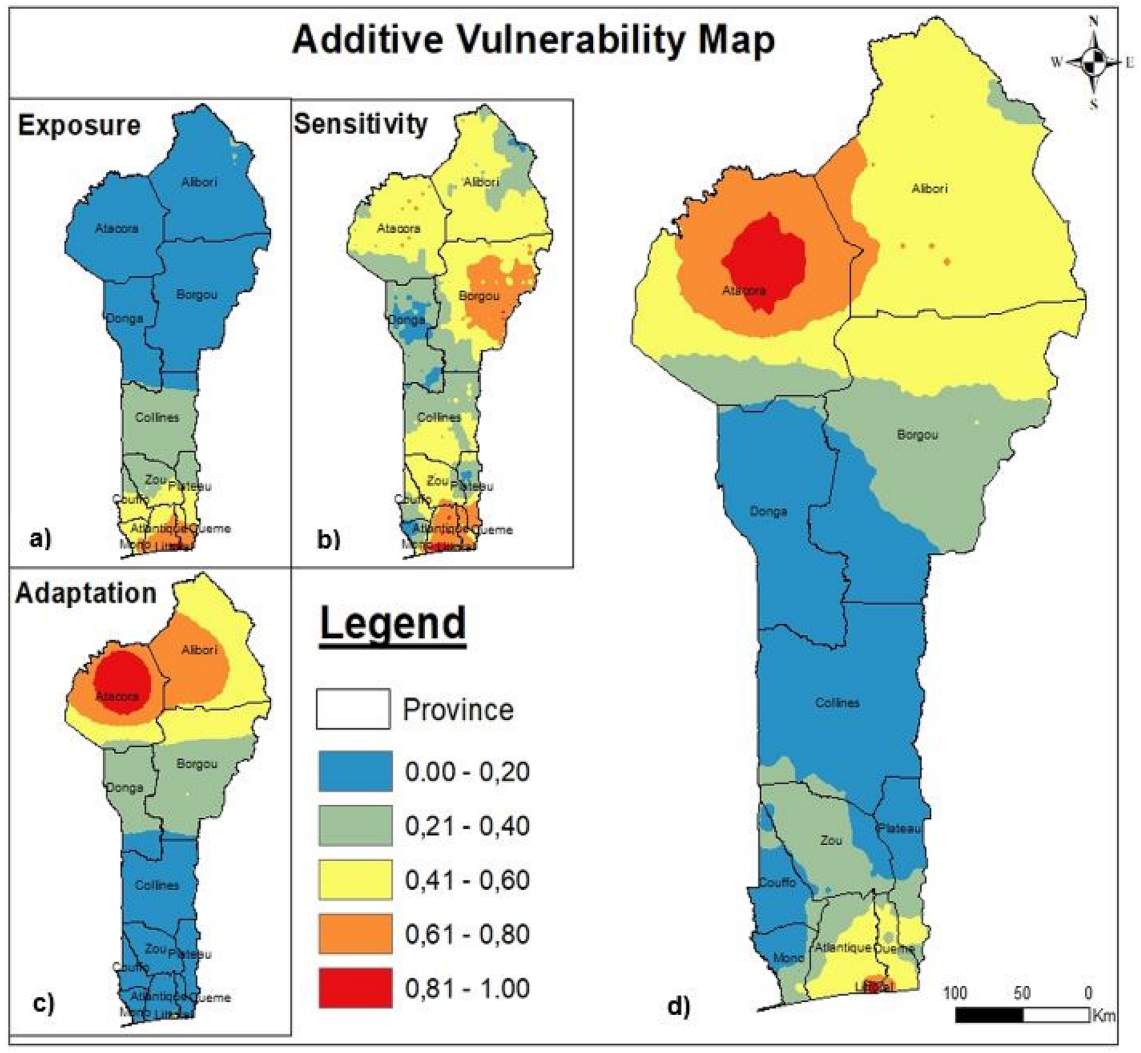
Additive vulnerability map.

The additive EVI (EVI_ad_) was considered a function of exposure, sensitivity and adaptation, as calculated in Eq. (6). Like the composite EVI (EVI_PCA_), as calculated by Eq. (6a) and shown in Fig. 3a, exposure also varied from north to south, with the lowest rate in the north and the highest in the south. As calculated by Eq. (6b) and shown in Fig. 3b, a high sensitivity rate was identified in the south, specifically in Atlantique, Littoral and Oueme provinces, and in the northeast, specifically, in eastern Borgou Province. Low-sensitivity areas were identified in the center of Donga, Plateau, Mono and Couffo provinces. As calculated by Eq. (6c) and shown in Fig. 3c, the adaptation rate was high in the center of Atacora Province and its surroundings, moderate in southern Benin (Littoral) and potential throughout central Benin.

To summarize, the EVI_ad_ vulnerability shown in Fig. 3d was high in Littoral and central Atacora, moderate in Atacora, low in Alibori, Atalantique and Oueme, slight in Borgou, Zou and potential in Collines, Donga, Mono, Couffo and Plateau. The highest additive EVI values were more closely related to the extreme exposure and adaptation values than to the sensitivity values in southern Benin^41^. This showed that additive EVI was influenced in decreasing order by adaptation, exposure and sensitivity. Therefore, we deduced that the areas with high fire frequencies were the most vulnerable. Sensitivity can therefore be perceived as an expression of resistance that had no influence on exposure^30^.

## Synthesis

**Figure 4:**
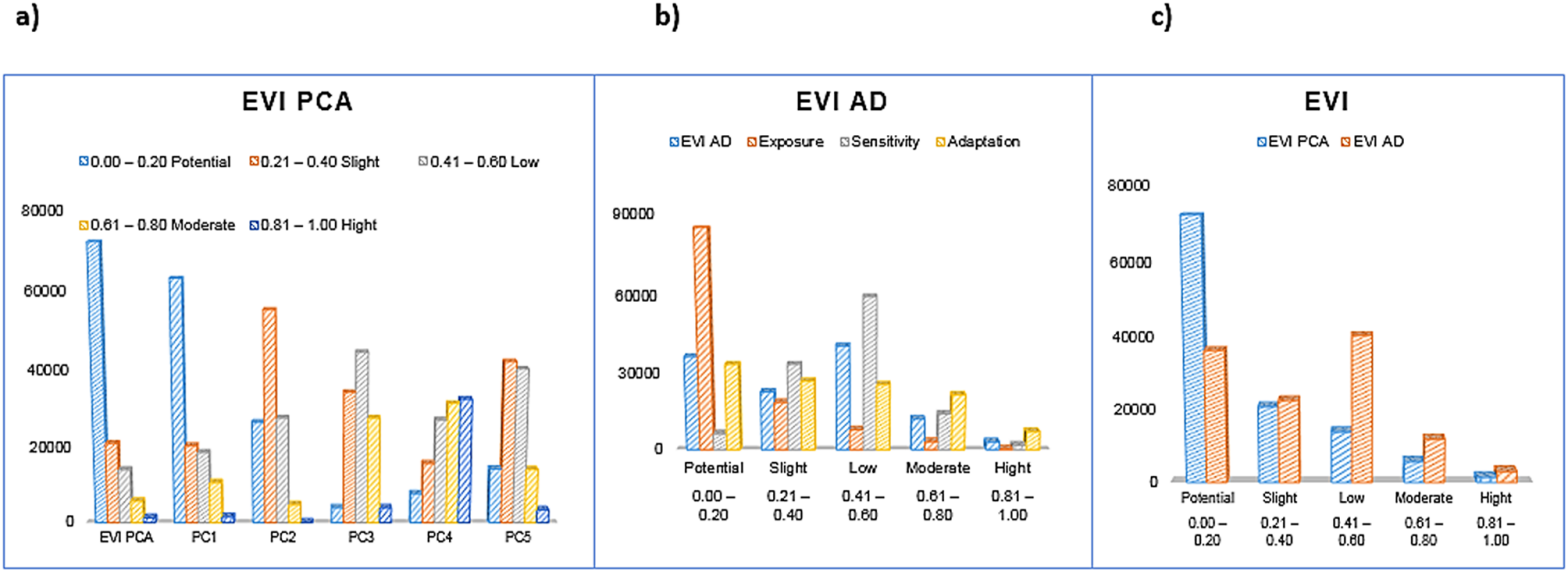
Classification areas quantification.

In the composite system, southern Benin was determined to be more vulnerable than northern Benin, but this trend was not observed in the additive system. However, Littoral Province was always determined to be vulnerable, regardless of the system. The different classified areas are shown in Fig. 4 both for the composite EVI and its components as well as the additive EVI and its components. Figure 4a shows the EVI PCA, Fig. 4b shows the EVI_AD_ and Fig. 4c shows both EVIs.

**Figure 5:**
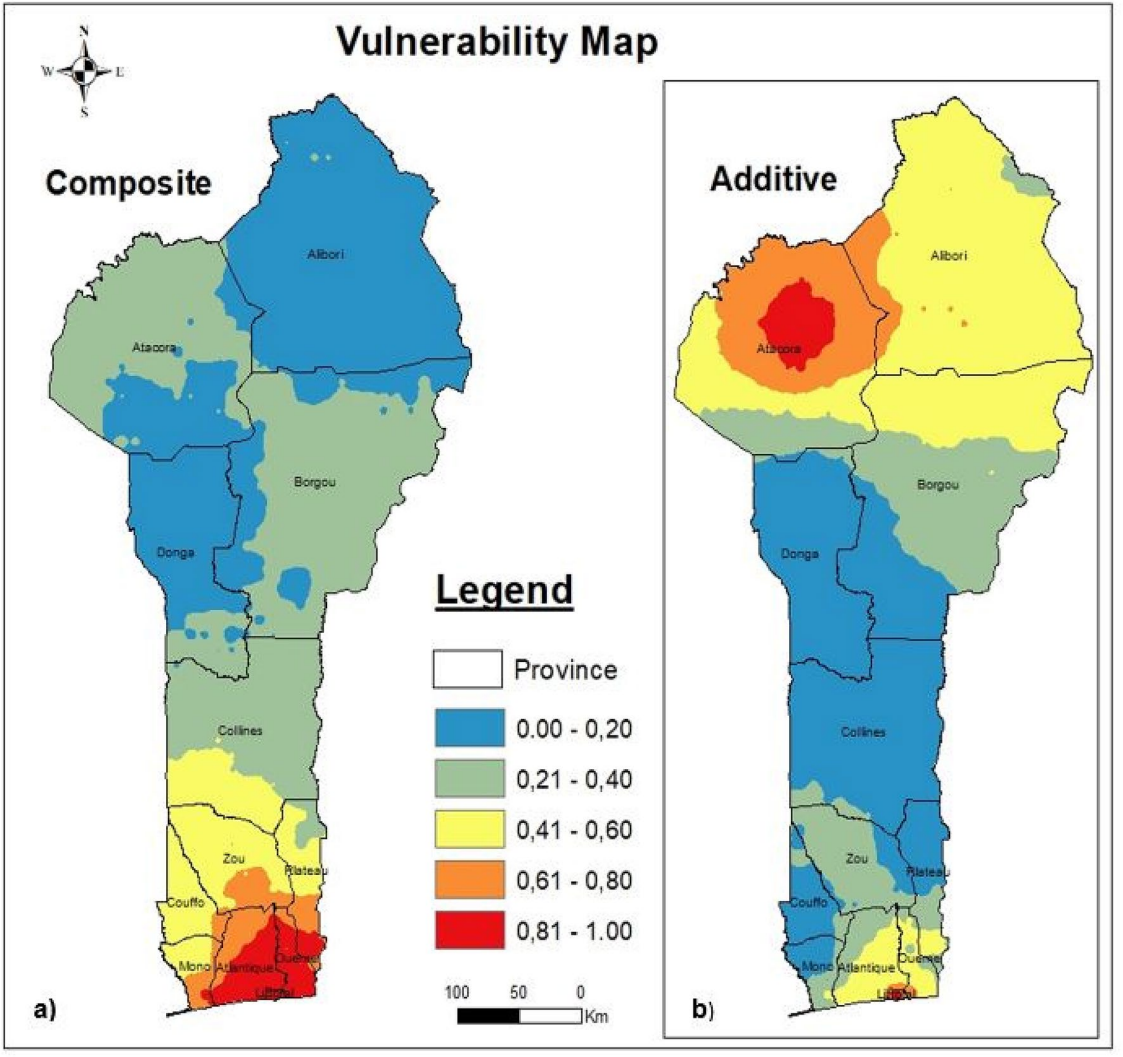
Observed composite and additive EVI map.

The results synthesis indicated that the composite EVI classified more areas as potentially and highly vulnerable than the additive EVI, i.e., 48,600 km^2^ and 3729 km^2^ for the EVI_PCA_ and 36,450 km^2^ and 3007 km^2^ for the EVI_ad_, respectively. The EVI_PCA_ values were 12,150 km^2^ higher for the potential vulnerability area and 722 km^2^ higher for the high vulnerability area than the EVI_AD_ values. Figure 5 shows both EVIs, a) EVI_PCA_ and b) EVI_ad_.

*Note* For the legend, the scale^32,41^ used is 0–1, from the lowest EVI level (0) to the highest EVI level (1).

### Similarity and dissimilarity

Given the discrepancies between the two EVI systems (Fig. 5), to improve the credibility of our study systems, we assessed the connection between the different components by calculating Moran’s index I and the coefficient of correlation between each constituent of the two EVIs.

#### Spatial autocorrelation using Moran’s I

Spatial autocorrelation^43^ measures the relationship among variable values according to the spatial arrangement of their values. Moran’s I^25^ is a correlation coefficient that measures the overall spatial autocorrelation of the data set by determining by how statistically similar one object is to the others surrounding it. The values we obtained were I_PCA_ = 0.955256 and I_AD_ = 0.989222. In addition, we performed a cluster analysis. Cluster analysis identifies statistically significant spatial clusters of high values (hot spots) and low values (cold spots) and provides confidence levels for each feature in the input feature class. Cold spots include elements of less importance, while hot spots include those of great interest that require special attention^44^. The composite EVI hot spot analysis^37^ notably varied from south to north, with a confidence of 99% in southern Benin and no significant values from the center to the north. In other words, southern Benin was a cluster of hot spots. Regarding the additive EVI, there was a cluster of hot spots in Littoral and Atacora. Only the Littoral confidence value was 99%, followed by Atacora at 95% confidence; the rest of the values were not significant. Then, in the additive system, there were a cluster of hot–cold spots in the south, cold-cold spots in the center and hot spots in the north.

#### Pearson’s correlation

This study used Pearson’s correlation coefficients to evaluate the relationship between each pair of components, which was useful for the automatic linear modeling regression. This calculation detects the presence or absence of a linear relationship between two continuous quantitative variables, i.e., it is a measure of the strength of the association between the two variables. The results are displayed in Table 5.

**Table 5:** Pearson correlation coefficient. *Note* EVI_Ad_ = additive ecological vulnerability index, EVI_PCA_ = composite ecological vulnerability index, Adap = adaptation, Sens = sensitivity and Expo = exposure and PC = principal component.

Components	EVI_Ad_	Adap	Sens	Expo	EVI_PCA_	PC6	PC5	PC4	PC3	PC2	PC1
EVI_Ad_	*1.000*	**0.860**	0.517	− 0.102	− 0.161	− 0.310	0.072	0.733	0.159	**0.787**	− 0.556
Adap	**0.860**	*1.000*	0.138	− 0.524	− 0.602	− 0.337	0.074	0.559	− 0.099	**0.814**	− **0.866**
Sens	0.517	0.138	*1.000*	0.157	0.416	0.180	0.172	0.672	0.202	0.229	0.060
expo	− 0.102	− 0.524	0.157	*1.000*	**0.844**	− 0.066	− 0.144	− 0.143	0.535	− 0.291	**0.801**
EVI_PCA_	− 0.161	− 0.602	0.416	**0.844**	*1.000*	0.016	0.109	− 0.121	0.573	− 0.306	**0.881**
PC6	− 0.310	− 0.337	0.180	− 0.066	0.016	*1.000*	− 0.454	0.154	− 0.223	− 0.663	0.182
PC5	0.072	0.074	0.172	− 0.144	0.109	− 0.454	*1.000*	− 0.239	− 0.112	0.384	0.048
PC4	**0.733**	0.559	0.672	− 0.143	− 0.121	0.154	− 0.239	*1.000*	− 0.139	0.308	− 0.385
PC3	0.159	− 0.099	0.202	0.535	**0.573**	− 0.223	− 0.112	0.139	*1.000*	0.199	0.327
PC2	**0.787**	**0.814**	0.229	− 0.291	− 0.306	− 0.663	0.384	0.308	0.199	*1.000*	− 0.669
PC1	− 0.556	− **0.866**	0.060	**0.801**	**0.881**	0.182	− 0.048	− 0.385	0.327	− 0.669	*1.000*

In summary, there was a strong positive correlation between the composite EVI (EVI_PCA_) and the first principal component, PC1 (0.881), exposure (0.844) and the third component, PC3 (0.573). Thus, we deduce that EVI_PCA_ is influenced by precipitation, temperature, relative humidity, sunlight and flooding. Regarding the additive EVI, EVI_ad_, there was a relatively strong correlation with adaptation (0.860), the second principal component, PC2 (0.787), and the fourth principal component, PC4 (0.733); consequently, we can deduce that GDP and population influence this EVI more than other factors.

Since the number of variables was higher than 3, we decided to use a linear regression to evaluate the degree of correlation between the variables; see Table 6 for the results. The results revealed that the correlation degree among the first three elements (PC1, exposure, and PC3) was higher than that among the latter three elements. (adaptation, PC2, and PC4).

**Table 6:** Linear regression coefficient.

Variables	EVI_PCA_	EVI_Ad_	R	R^2^
PC1	0.881	–	0.991	0.830
Expo	0.844	–		
PC3	0.573	–		
Adap	–	0.860	0.873	0.761
PC2	–	0.787		
PC4	–	0.733		

## Discussion

According to^7^, identifying fragile ecological areas is imperative for ecological protection and environmental organization and management. Therefore, assessing ecological vulnerability is crucial for the study of ecosystem vulnerability^45^. Based on the current conditions and previous predictions, the EVI was classified from the lowest vulnerability (potential) to the highest vulnerability (high), as shown in Table 4. Overall, this study obtained three main results, which are highlighted below.

The first result concerned the spatial variation in EVI. In the composite system, the EVI (EVI_PCA_) varied from north to south, with Littoral being a vulnerable province and Alibori being a stable province. In the additive system, EVI (EVI_ad_), both southern and northern Benin were identified as vulnerable, especially northern Benin, and Littoral (which was identified as vulnerable by the composite system) and central Atacora (which was identified as potentially vulnerable by the composite system), respectively, were identified as vulnerable.

The second result was the calculation of the spatial autocorrelation coefficients (Moran’s I) of each EVI, which were I_PCA_ = 0.955256 and I_AD_ = 0.989222 for the composite and additive systems, respectively. Both of these values are very high and are better than those reported in^46^. Although the spatial variations in these systems were obviously different, their Moran’s I values remained very high. However, according to Moran’s I, the spatial autocorrelation of the additive system was higher than that of the composite system. The principal component analysis approach assumes no prior relationship between the different factors and allows their relationships to develop from the statistical analysis, thus indicating the regional spatial variability of the components^8^. The observed discrepancies in spatial variation outcomes did not mean that there was a lack of spatial organization between the components. Therefore, graphic dissimilarities (differences in spatial distributions) do not challenge the spatial layout of the components or notably, their correlations.

The third result was from the cluster analysis, showing high-high clusters in the south for the composite system and in the north for the additive system. We deduce that regardless of the system used to calculate vulnerability, ecosystems in central Benin are still relatively stable. Central Benin has a moderate population density and moderate soil organic carbon levels. Littoral has a high population density rate, while Borgou has a high soil organic carbon level. These outcomes reveal that southern Benin is seriously threatened according to the composite system and that northern Benin is seriously threatened according to the additive system. These findings were explained and discussed with reference to available studies.

We used IDW interpolation, as opposed to^41^, who used kriging interpolation. We note that the indicators used in that study were slightly different from those in this study and were not classified similarly; in addition, different analysis assumptions were applied. His results show a strong positive correlation between sensitivity and the additive EVI (EVI_AD_), which is slightly different from the results of our study. In this study, we found a moderate correlation between these two factors. This difference in the outcomes can be attributed to the difference in the indicators and their distribution in the system. Nonetheless, that study showed that additive vulnerability is primarily influenced by adaptation, exposure and sensitivity; our study led us to put these elements in the order of adaptation, sensitivity and exposure. Both studies placed adaptation in the same position. Although the considered variables were different, we reached the same conclusion regarding adaptation, which can be considered a strength of our additive system.

Densely populated areas were determined to be very vulnerable^47^. High sensitivity rates were detected in southern Benin, including in Littoral, Atlantique, and Oueme. Housing and density indicators were classified as sensitivity variables, which means that density is still a threat to ecosystem stability. Littoral Province, the economic capital of Benin, which has the highest population density (more than 8000 inhabitants per square kilometer, according to the averaged raw data), and Atlantique and Oueme provinces, newly developed residential areas, were classified as extremely vulnerable. Alibori Province, the largest and least populated province, was classified as the most stable area in the composite system. We can deduce from this analysis that the population density also has a great impact on the composite system. In the additive system, Littoral remained an extremely vulnerable area, and central Atacora and Collines were the most stable areas. This outcome confirms that density in Littoral is a serious challenge to stability according to both systems.

However, the composite system than the additive system is more credible since it is based on SPSS, a statistical software, and is therefore empirical. In contrast, the additive system can be unreliable, since the indicators, as a whole, are classified according to the user. This classification method is subjective, and therefore theoretical (here, we based our indicators on expert advice and IPCC recommendations); hence, it leaves room for doubt. This study found that coastal zones, i.e., Littoral, are the most vulnerable^33,34,48^. This finding indicates the reality for our study. The extremely vulnerable areas identified by the composite system were high per capita density areas, which emphasized that density was a decisive indicator in our composite system. This analysis uncovered significant spatial variation in population vulnerability in southern Benin. According to the raw data we collected, the average density per capita in Borgou is 35.909%, while in Littoral, it is 8003.636%, i.e., 223 times higher than that in Borgou. Borgou is made up of several communes, while Littoral consists only of Cotonou, the economic and administrative capital of Benin, which is a highly desirable area. The demand for buildings has forced people to occupy some natural drainage channels, making this commune vulnerable to flooding. Southern Benin is less spacious but has more inhabitants than northern Benin because almost the entire administrative system of the country is located there, as well as one of the largest markets in West Africa. There is a need for an efficient decentralization process according to the determined standards. Our study revealed that regions with lower density per capita were the least vulnerable.

The additive system found that the areas with high bush fires and soil organic carbon rates were the most vulnerable. Thus, vulnerability is specific to the context^34^, since the factors that make a region or a community vulnerable can vary among different regions and community. The vulnerability of the northern area that was highlighted by the additive system can be explained by the practice of intensive agriculture (soil organic carbon) and the bush fires involved in these practices. Northern Benin is an agricultural area, and cotton cultivation is common; hence, there are high levels of pesticide use. Agriculture is very important for the Beninese economy and hence pesticides are used. Vulnerability in southern Benin is related to climate, flooding, and the high population density, while vulnerability in northern Benin is related to bush fires and soil organic matter levels. Although the systems and indicator groupings were different, they reached the same conclusion about Littoral Province. In the additive system, the vulnerable areas corresponded to areas with high soil organic carbon.

It is important to point out that this study suffers from certain limitations^38^. For example, data for all the indicators from the same time period were not always available, some required data were inaccessible and some data were gathered from the public domain. This can be interpreted as a weakness of our system. Since public-domain data are not accurate, they can result in biased outputs, which should not be ignored. The determined spatial and temporal variation, as well as the type of degradation under consideration, depends on the input data sets for the analysis and modeling^39^. Using automatic linear modeling model building (ALMMB), our results were improved.

**Figure 6:**
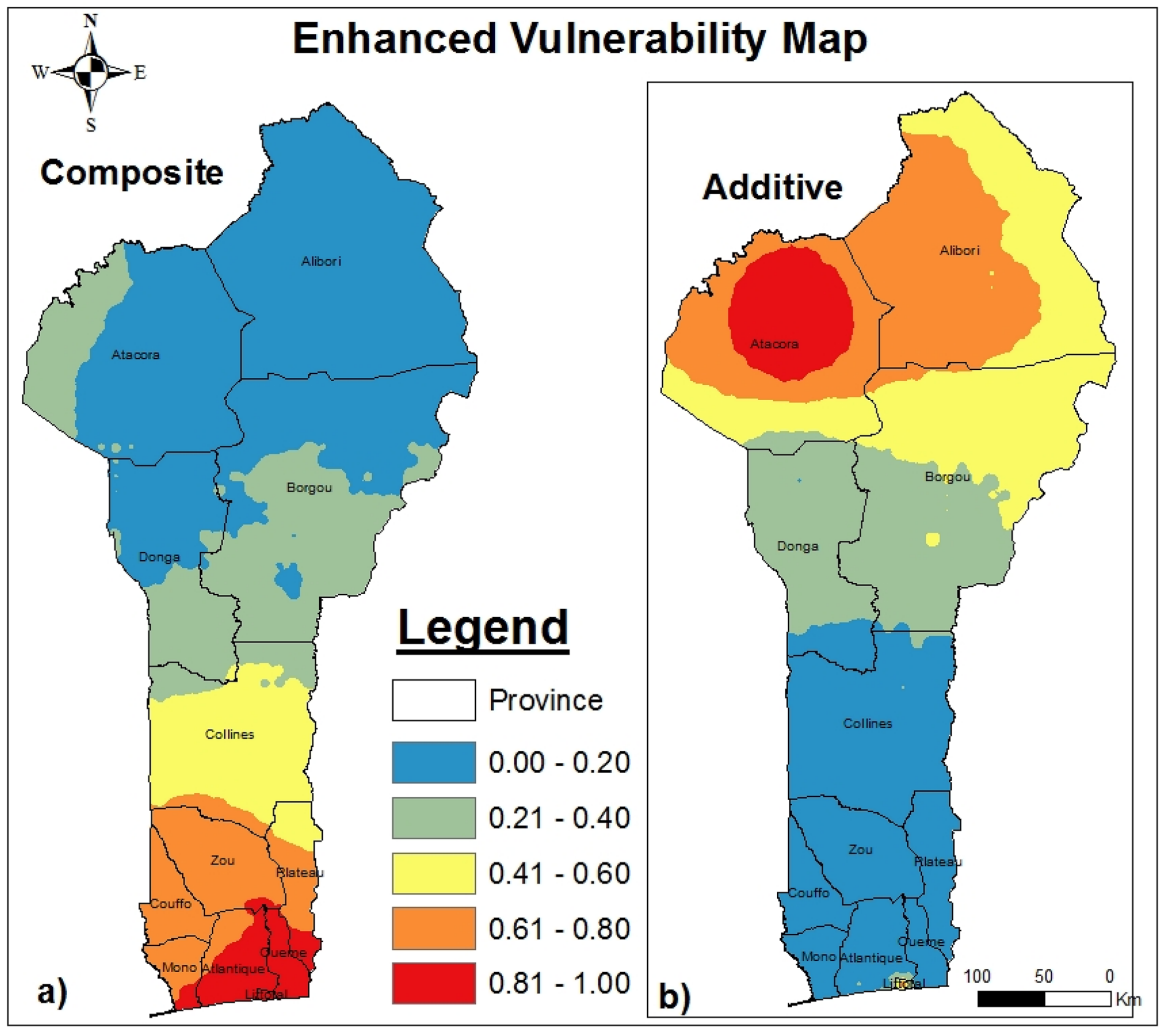
Improving composite and additive EVI map.

The main objective of automatic linear modeling model building (ALMMB) was to improve the present study outcomes by enhancing the accuracy of the established system based on the adjusted chi-square Pearson correlation. Using automatic linear modeling regression combined with the best subsets method in SPSS 23, we tried to enhance each observed vulnerability level. Table 7 displays both the observed and enhanced rates for each EVI, and Fig. 6 displays the map of the enhanced values. We note that the potentially vulnerable areas^32^ increase or decrease in size less than the highly vulnerable areas.

**Table 7:** Observed and enhanced rate for EVI.

EVI		EVI_PCA_					EVI_AD_		
Component	PC1	PC2	PC3	PC4	PC5	PC6	Expo	Sens	Adap
Observed (Obs.)	0.446	0.159	0.123	0.103	0.093	0.076	0.340	0.330	0.330
Enhanced (Enh.)	0.730	0.050	0.200	0.010	0.010	0.000	0.010	0.190	0.800

**Figure 7:**
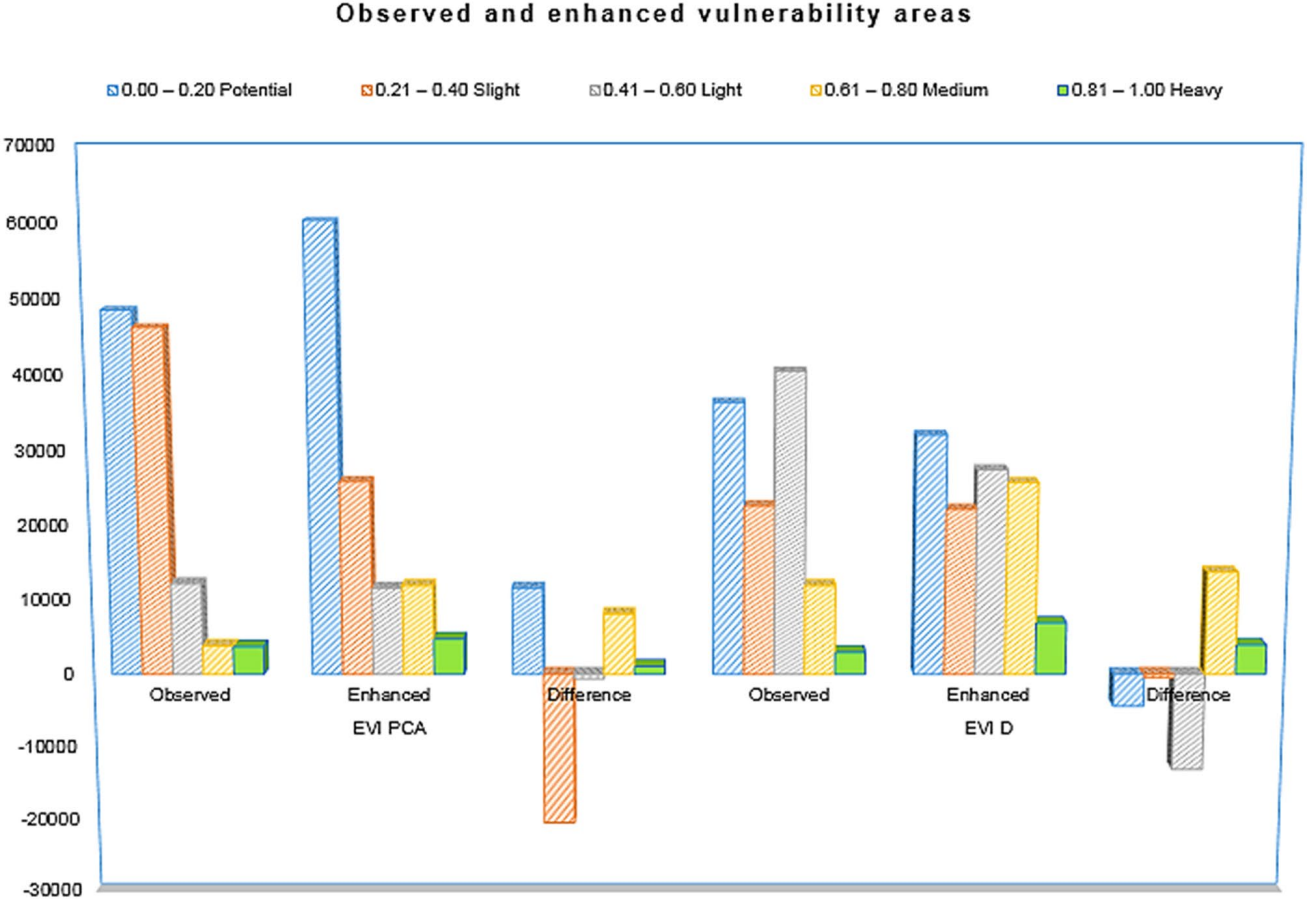
Synthesis of different classified areas.

Based on Table 8, in the composite system, increases in both the potentially and highly vulnerable areas were highlighted. The observed potentially vulnerable area was 48,600 km^2^, and the enhanced potentially vulnerable area was 60,269 km^2^. The observed highly vulnerable area was 3729 km^2^, and the enhanced highly vulnerable area was 4812 km^2^; the differences in these values were 11,669 km^2^ and 1083 km^2^, respectively. A decrease in the potentially vulnerable area and an increase in the highly vulnerable area were noted in the additive system. In the additive system, the observed potentially vulnerable area was 36,450 km^2^, and the enhanced potentially vulnerable area was 32,119 km^2^, for a difference of 4331 km^2^. The observed highly vulnerable area was 3007 km^2^, and the enhanced highly vulnerable area was 6977 km^2^, for a difference of 3970 km^2^, i.e., more than the double the observed value. However, according to the enhanced composite model, much attention should be paid to all southern provinces, especially Zou, Oueme and Plateau. Figure 6 displays the enhanced vulnerability mapping for a) the composite system and b) the additive system. Figure 7 summarizes the different classified areas and their differences.

**Table 8:** Observed and enhanced vulnerability areas. *Note* Classif. = classification, Dif. = difference, Qualif. = qualification, Inc. = increase and Reg. = regression.

Rank	Classif	EVI_PCA_				EVI_AD_			
		Obs. (km^2^)	Enh. (km^2^)	Dif. (km^2^)	Qualif	Obs. (km^2^)	Enh. (km^2^)	Dif (km^2^)	Qualif
0.00–0.20	Potential	48,600	60,269	11,669	Inc	36,450	32,119	− 4331	Reg
0.21–0.40	Slight	46,314	25,984	− 20,330	Reg	22,736	22,255	− 481	Reg
0.41–0.60	Low	12,270	11,669	− 601	Reg	40,540	27,548	− 12,992	Reg
0.61–0.80	Moderate	3849	12,030	8180	Inc	12,030	25,864	13,834	Inc
0.81–1.00	High	3729	4812	1083	Inc	3007	6977	3970	Inc

In summary, the composite system was vulnerable to climate and flooding (and to some extent to population density as well, as in Littoral), while the additive system was vulnerable to bush fires and soil organic matter. Littoral was identified as a vulnerable area in both systems. Finally, to improve the accuracy of our results, we used ALMMB. The results showed both increases and decreases in the size of vulnerable areas. The present study used a combination of GIS, PCA and ALMMB to accurately assess the vulnerability of terrestrial ecosystems in Benin.

## Conclusion

The challenge of ecological vulnerability assessment is to identify vulnerable areas and propose optimal measures for their future management. In this study, the spatial variation in vulnerability differed according to the system considered (composite or additive). Regardless of the system, the Littoral Province was always shown to be vulnerable; this is likely due to its geographical position (coastal), which makes it subject to rainfall fluctuations, and its excessive density. Donga was identified the most stable province. Density hotspots related to high-population areas are threatened ecosystems in Benin. Applying different systems of analysis does not exclude the possibility of similarities in their outputs since, mathematically speaking, they all belong to the same domain of definition and are materialized here by the same analytical indicators.

Special attention should be paid to the ecosystem in Littoral. This will involve initiating a study that should lead to the establishment of a master plan^49^, which must propose rigorous measures based on the issues faced in this province. The extremely vulnerable areas revealed by the composite system are the high per capita density areas, which shows that population density is a crucial factor in our composite system. Southern Benin is less spacious than northern Benin is but more populated because almost the entire administrative system of Benin, as well as one of the largest markets in West Africa, is located there. If the administration of Benin were decentralized, many officials would have to move; this would allow residents to stop living in drainage areas, and the water might find its natural flow channel. The additive system identified areas with high bush fire rates as the most vulnerable. Thus, vulnerability is specific to the context^34^, since what makes a region vulnerable can differ from region to region. In southern Benin, population density and climate were threats, while in northern Benin, soil organic matter was the source of vulnerability. This study promoted the use of both GIS and statistical methods to accurately identify and account for fragile ecological areas.

This study quantitatively and accurately evaluated the ecological environmental quality of terrestrial ecosystems in the Republic of Benin under different scenarios. The vulnerability of Benin’s terrestrial ecosystems was confirmed, and the vulnerable areas were clearly identified. These ecosystems, which are prone to instability, will thus require more attention in the future. We expect that this study will assist in obtaining funding to support research to maintain sustainability in the developing country of Benin and will be helpful for making policy decisions.
